# Mpox and the need for improved diagnostics – correspondence

**DOI:** 10.1097/MS9.0000000000000436

**Published:** 2023-04-04

**Authors:** Shailesh K. Patel, Jigyasa Rana, Aditya Agrawal, Nikhil K. Channabasappa, Ankush K. Niranjan, Talha B. Emran

**Affiliations:** a Department of Veterinary Pathology; b Department of Veterinary Microbiology, College of Veterinary Science and Animal Husbandry; c Department of Veterinary Physiology and Biochemistry, College of Veterinary Science and Animal Husbandry, Rewa, Madhya Pradesh; d Department of Veterinary Anatomy, Faculty of Veterinary and Animal Sciences, Rajiv Gandhi South Campus, Banaras Hindu University, Barkachha, Mirzapur, Uttar Pradesh, India; e Department of Pharmacy, BGC Trust University Bangladesh, Chittagong; f Department of Pharmacy, Faculty of Allied Health Sciences, Daffodil International University, Dhaka, Bangladesh

*Dear Editor*,

Amid the global challenge of coronavirus disease 2019 (COVID-19), the recently emerged monkeypox (Mpox) has added another burden to global healthcare facilities. The Mpox virus is a double-stranded DNA virus that affects humans and animals. As of 7 March 2023, a total of 86 369 confirmed cases with 108 deaths from 110 countries/areas/territories were reported (https://worldhealthorg.shinyapps.io/mpx_global/#section-fns2). Most of the cases of this multination outbreak of Mpox have been reported in locations that have historically been free from the infection, thereby highlighting the fact that rapid and accurate diagnosis of Mpox is necessary for effective surveillance of the disease. The diagnostic capacity against any emerging or reemerging disease is a crucial part of public health preparedness, and inadequate diagnostic capacity illustrates the consequences of the ongoing worldwide spread of Mpox.

Mpox exhibited a great deal of syndromic diversity, from asymptomatic infection to fever, confined skin lesions, and disseminated rash^1^. In addition, plenty of Mpox cases presented with only genital lesions, which was inconsistent with earlier findings in the USA and Sub-Saharan Africa. Therefore, solely considering the syndromic cluster investigation could misdiagnose Mpox as other genital infections, allowing the unchecked transmission of Mpox^2^. For confirmatory Mpox diagnosis, the samples such as swabs of the exudates, the lesion surface, roofs from conglomerate lesions, and crusts from cutaneous lesions, must be properly collected and dispatched to a laboratory with all the necessary diagnostic facilities. The majority of Mpox diagnosis relies on conventional PCR, DNA sequencing, and real-time PCR. Therefore, for accurate and timely diagnosis, samples should be packaged and transported as soon as possible using recommended protocols^3^. Additionally, Mpox must be differentially diagnosed with measles, chickenpox, cutaneous bacterial infections, syphilis, scabies, and allergies. Lymphadenopathy can prove crucial in distinguishing Mpox from smallpox and chickenpox at the prodromal stage^4^.

Molecular techniques such as PCR, real-time PCR, genome sequencing, LAMP (loop-mediated isothermal amplification), CRISPR (clustered regularly interspaced short palindromic repeats), etc. are sensitive and can efficiently diagnose Mpox. Presently, these tests are working best in major laboratories, but their application as a real-time diagnostic tool in remote village areas is very limited owing to a lack of resources. Moreover, a PCR test is generally erroneous since the viremia lasts for a short span of time in relation to the time specimens are generally collected after symptoms begin. The traditional techniques of cell culture, virus isolation, and electron microscopic examination still remain valid, but they require sophisticated laboratories and highly skilled personnel. The immunological assays can be used for the identification of cases retrospectively, but their cross-reactivity with other orthopoxviruses is a major limitation. For patients who have already been exposed to the orthopoxviruses, even through immunization, anti-orthopoxvirus immunoglobulin G (IgG) alone is not sufficient to diagnose the infection. However, tests evaluating the anti-orthopoxvirus IgM are more convenient for the diagnosis of recent retrospective infections, including in immunized individuals, but the false negatives and false positives outweigh their advantages. The diagnostic tests that depend on samples being collected by healthcare workers have more limitations owing to the social stigma of sexually transmitted diseases.

The little progress in the development of point-of-care testing (POCT) against Mpox, which can be globally deployed in the field without any assistance from healthcare personnel, is another constraint. Recently, a pilot study of the Tetracore Orthopox BioThreat Alert revealed that vaccinia and Mpox viruses could be accurately detected in serum samples. Despite not being specific for the Mpox virus, this assay can be used for the detection of orthopoxviruses by proxy in areas endemic to Mpox. Recently, the WHO recommended nucleic acid amplification testing (NAAT) for the detection of the Mpox genome. However, the development of diagnostic assays that can be performed by an untrained individual in very basic settings is crucial for the containment of Mpox since Mpox patients often seek prompt diagnosis and primary healthcare in rural settings without electricity.

In conclusion, PCR still remains the mainstay of Mpox testing, but the development of easily available, even lower sensitivity diagnostic assays should also be considered as a substitute for PCR. Furthermore, advances in innovative approaches and technologies developed during the COVID-19 pandemic, such as microfluidics, CRISPR‐based POCT, the Virolens system, wastewater surveillance, and biosensors, could be customized for Mpox diagnosis^5^. Finally, the integration of both phenotypic and genotypic assays for Mpox detection and characterization is crucial for an adaptive public health response.

## Ethical approval

Not applicable.

## Consent

Not applicable.

## Sources of funding

None.

## Author contribution

All the authors substantially contributed to this work.

## Conflicts of interest disclosure

None declared.

## Provenance and peer review

Not commissioned, externally peer-reviewed.
